# Epicardial Ventricular Tachycardia Ablation: A Contemporary Review of Indications, Techniques, and Practical Approaches for Challenging Substrates

**DOI:** 10.31083/RCM44332

**Published:** 2026-01-23

**Authors:** Maiko Kuroda, Kenichi Hiroshima, Kenji Ando

**Affiliations:** ^1^Department of Cardiology, Kokura Memorial Hospital, Kitakyushu, 802-8555 Fukuoka, Japan

**Keywords:** ablation, epicardial, ventricular tachycardia, mapping

## Abstract

Epicardial ablation is an increasingly important treatment for refractory ventricular tachycardia (VT), particularly in nonischemic cardiomyopathy or when the substrate is epicardial or mid-myocardial. This review provides an overview of the pathophysiology and disease-specific characteristics of VT substrates, with a particular focus on epicardial involvement and indications based on electrocardiographic, imaging, and clinical findings. We present advanced substrate-mapping strategies, including functional and high-resolution approaches, and practical examples of three-dimensional mapping using the CARTO™ (Biosense Webster, Diamond Bar, CA, USA) and EnSite™ (Abbott, Abbott Park, IL, USA) systems to overcome the known limitations of conventional mapping techniques. In the latter part of the review, we discuss the technical aspects of epicardial access, as well as the clinical challenges and strategies for challenging scenarios, such as bipolar ablation and ablation after prior cardiac surgery, supported by practical examples from our institution. We also highlight future perspectives. These insights are expected to contribute to the optimization of treatment strategies for refractory epicardial VT and to support the development of more precise and durable patient care.

## 1. Introduction

Ventricular tachycardia (VT) is a life-threatening arrhythmia that occurs in patients with structural heart disease, and recurrent VT, in particular, is associated with a high risk of sudden cardiac death and severe hemodynamic compromise, underscoring the critical importance of timely therapeutic intervention. In recent years, catheter ablation has become the mainstay of VT management, and it has been reported to not only alleviate symptoms but also potentially improve long-term outcomes in selected disease conditions [1]. In ischemic cardiomyopathy (ICM), the scar tissue is primarily located in the subendocardial layer, with relatively well-defined structural features, making traditional mapping strategies and ablation techniques generally more effective. In contrast, nonischemic cardiomyopathy (NICM) often involves a heterogeneous scar distribution, with lesions extending into the mid-myocardial and epicardial layers, where endocardial ablation alone frequently fails to achieve a sufficient therapeutic effect [2]. Given this background, epicardial ablation has emerged as an important therapeutic option for patients with refractory VT.

In 1996, Sosa *et al*. [3] developed a pioneering technique for percutaneous epicardial access and successfully performed mapping and ablation of VT in patients with Chagas cardiomyopathy, a disease in which VT often arises from epicardial substrates. When the arrhythmogenic substrate cannot be reached by standard endocardial approaches, epicardial ablation becomes an essential procedure, although it carries specific risks not typically seen with endocardial ablation, such as pericarditis and coronary artery injury, thereby requiring a high level of operator skill and experience to ensure safety and effectiveness.

In recent years, the continued success of VT ablation has been driven by advances in mapping technologies. This progress is largely attributable to the evolution of substrate mapping and substrate-targeted ablation strategies. Because VT circuits are formed within thick layers of ventricular myocardium and possess inherently three-dimensional structures, conventional two-dimensional mapping techniques often provide insufficient information. Accurate visualization of the VT substrate is therefore becoming increasingly important for improving ablation outcomes and preventing recurrence.

This review focuses on the epicardial mapping and ablation strategies in the treatment of refractory VT. Section 2 explains when an epicardial approach is needed and how it can be performed safely. It describes electrocardiogram (ECG) signs, pericardial puncture, and how to prevent and manage complications. Section 3 outlines the pathophysiology of VT and shows how different diseases shape epicardial substrates and the need for ablation. Section 4 reviews conventional mapping methods and explains how they guide the decision for an epicardial approach. Section 5 presents newer substrate-mapping techniques and shows practical ways to visualize three-dimensional circuits. Section 6 deals with difficult cases, such as epicardial ablation after open-heart surgery or deep substrates, and explains when strategies like bipolar ablation are helpful. Section 7 looks at future supportive technologies for epicardial ablation. Together, these sections provide a clear and practical guide to epicardial VT ablation, covering everything from indications and techniques to future perspectives.

## 2. Epicardial Access and Ablation

### 2.1 Predicting the Need for Epicardial Access Based on the Electrocardiogram

When epicardial VT is suspected, the 12-lead ECG provides a useful, noninvasive, and immediate clue. Along with the underlying heart disease described in section 2, both the morphological and interval criteria from the ECG are used to assess the need for an epicardial approach.

#### 2.1.1 Morphology Criteria

In epicardial VT, it is important to examine the initial QRS polarity (r or q wave) in leads facing the VT origin. When depolarization starts in the epicardium, the electrical vector moves away from the lead, often producing an initial Q wave or QS complex.

The following are typical patterns:

(1) Initial Q wave in lead I: suggests an epicardial origin in the basal or superior apical region of the LV [4].

(2) Q waves in leads II, III, and augmented vector foot (aVF): suggest an epicardial origin in the basal inferior LV [4].

If no Q waves are seen in II, III, and aVF: this may suggest a basal superior origin in the LV.

(3) Q wave in lead V2: may indicate an epicardial origin near the apex.

(4) Augmented vector right (aVR)/augmented vector left (aVL) ratio <1: suggests an epicardial origin in the basal inferior LV.

Morphology criteria are helpful for left ventricle (LV)-origin VT, but less reliable for right ventricle (RV)-origin VT [5].

#### 2.1.2 Interval Criteria

In epicardial VT, activation spreads slowly from the epicardium to the endocardium, reaching the subendocardial Purkinje fibers and then depolarizing both ventricles. As a result, the QRS upstroke is delayed.

Several ECG markers have been proposed to reflect this delay:

(1) Pseudo-delta wave ≥34 ms

(2) Intrinsicoid deflection time ≥85 ms (in lead V2)

The intrinsicoid deflection time is the time from the start of the QRS complex to the peak of the R wave on an ECG.

(3) RS interval ≥121 ms

(4) Maximum deflection index (MDI) ≥0.55

The MDI is the ratio of the time from the QRS onset to the peak deflection, divided by the total QRS duration on the ECG.

These criteria are particularly accurate for identifying epicardial VTs from the basal or lateral LVs. However, these markers are not reliable for VT originating from the RV or septum, so caution is needed [6].

### 2.2 Pericardial Puncture Technique

#### 2.2.1 Percutaneous Epicardial Access Procedure

The following 6 steps describe the actual puncture procedure.

Step 1: Preprocedural assessment.

CT is used to assess pericardial adhesions and determine feasible puncture directions. Right coronary angiography (and left, if needed) is performed to visualize the coronary artery.

Step 2: Puncture site selection.

The standard approach is from the left of the xiphoid and below the left costal arch.

After local anesthesia, start the puncture using a Tuohy needle with a contrast-filled syringe.

Step 3: Fluoroscopic and contrast confirmation.

In the anterior approach, the needle direction is checked in the anteroposterior (AP) or left lateral view. Once cardiac pulsation is felt, contrast is injected. In the posterior approach, confirmation is done in the right anterior oblique (RAO) view. Once contrast spreads in the pericardial space, insert a guidewire.

Step 4: Management of the RV puncture.

Rapid washout of contrast or aspiration of blood indicates an RV puncture. The needle should be pulled back a few millimeters into the pericardial space, and contrast injection should be repeated.

Step 5: Guidewire and sheath insertion.

After guidewire insertion, free advancement into the epicardial space is confirmed under left anterior oblique (LAO) fluoroscopy. Then, a sheath is inserted.

Step 6: Postprocedural care.

Methylprednisolone (0.5–1.0 mg/kg) is administered post-procedure to prevent pericarditis.

**Fig. 1.**
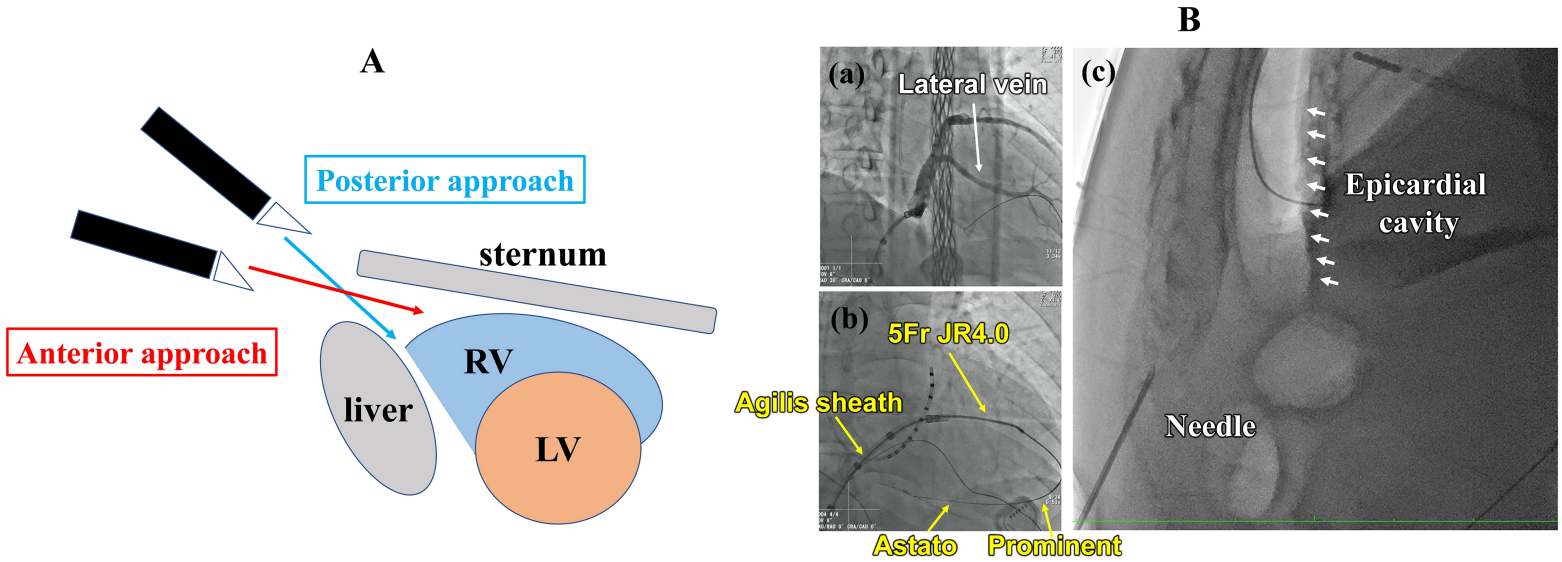
**Two approaches for the epicardial puncture and Epicardial puncture using the carbon dioxide injection method**. Fig. 1A. Schematic illustration of anterior and posterior epicardial puncture approaches. Epicardial access has two approaches: anterior and posterior, depending on the needle angle and direction. Please see section 5-2 for more details about each approach. LV, left ventricle; RV, right ventricle. Fig. 1B (a) The lateral vein of the coronary vein was confirmed by contrast injection. Fig. 1B (b) Using a diagnostic JR4.0 catheter, the lateral vein was selected. A microcatheter (Prominent®) and a 0.014-inch high-tip-load wire (Astato®) were used to intentionally perforate the distal branch and access the epicardial space. Fig. 1B (c) After removing the 0.014-inch wire, 100–150 mL of CO_2_ was injected through the microcatheter. The anterior pericardial space became visible under fluoroscopy. Since CO_2_ escapes quickly during the needle puncture, a guidewire should be inserted into the epicardial space immediately after the puncture.

There are two types of percutaneous epicardial puncture approaches: anterior and posterior, depending on the angle and direction of the needle insertion (see Fig. 1A). The anterior approach uses a shallow angle (20–30°) to insert the needle slightly below and toward the inside of the xiphoid process. It has the advantage of a lower risk of liver injury and easy access to the RV anterior wall, but care is needed due to possible interference with the back of the sternum and closeness to the internal thoracic artery. On the other hand, the posterior approach uses a steeper angle (30–45°) and advances the needle through Larrey’s gap, where the diaphragm muscle is very thin or missing. Larrey’s gap is a space between the xiphoid and diaphragm, located near the left parasternal area, bordered by the sternum in front, pericardium behind, and diaphragm below. Larrey’s gap is a space between the xiphoid process and the diaphragm. It is located on the left side near the sternum, with the sternum in front, the pericardium behind, and the diaphragm below. This approach gives access to areas with fewer pericardial adhesions and allows a wide reach to the posterior wall, but it has risks such as liver or diaphragm injury, and the needle tip may be hard to track under fluoroscopy due to overlap with the ribs, bowel gas, diaphragm, or spine. Access from the side opposite to the area of ablation (anterior or posterior) is usually recommended, but pericardial adhesions can sometimes make either approach difficult.

#### 2.2.2 Supportive Technique During the Puncture: Carbon Dioxide Insufflation

In recent years, injecting carbon dioxide (CO_2_) into the pericardial space via the right atrial appendage or coronary veins has gained attention as a support technique during epicardial access. It is mainly used to assist a subxiphoid puncture. In this method, CO_2_ spreads to the front of the pericardial space while the patient is lying down and at rest. This makes the pericardial space visible under fluoroscopy and creates a space in front of the heart, allowing safe access to the pericardial cavity while avoiding accidental injury to the coronary arteries or myocardium (Fig. 1B). The CO_2_ injection into the pericardial space from the coronary sinus is performed as follows. A coronary vein branch is selected using a diagnostic JR4.0 catheter. Then, a microcatheter and a 0.014-inch high-tip-load wire are used to intentionally perforate the distal part of the vein and access the epicardial space. After removing the 0.014-inch high-tip-load wire, 100–150 mL of CO_2_ is slowly injected through the microcatheter. This makes the anterior pericardial space visible under fluoroscopy and allows a safe and reliable subxiphoid puncture with a needle [7] (Video 1). In the Epi-CO_2_ Registry, the procedure was successful in 101 of 102 cases, with no major complications, showing it is a safe and reproducible technique [8]. To reduce bleeding, the wire should be advanced through a low-pressure site—ideally, the peripheral part of the vein. Smaller branches are safer for hemostasis, but if not available, another branch should be chosen. If an LV lead has already been implanted, another coronary sinus branch should be used to avoid the risk of lead dislodgement. There is a risk of accidentally entering a vein that goes into the myocardium. In that case, the wire will not reach the epicardial space, so it is important to check the wire tip’s position and movement under fluoroscopy. After advancing the wire into the epicardial space, a small amount of contrast is injected through the microcatheter to rule out pericardial adhesions. CO_2_ is then slowly injected [9]. During the CO_2_ injection, the blood pressure rarely drops. Because CO_2_ is about 20 times more soluble in water than air, it does not remain in the epicardial space for long. This minimizes noise on the electrocardiogram and is unlikely to interfere with mapping [10]. This technique may lower the risk of complications seen with a conventional epicardial puncture and could improve the safety, especially in centers with less experience in epicardial ablation [11].

**Video 1.** **Carbon dioxide–assisted epicardial puncture**. Representative video demonstrating CO_2_ injection and visualization of the pericardial space. Video associated with this article can be found in the online version at https://doi.org/10.31083/RCM44332.

#### 2.2.3 Recent Technical Refinements for Safer Epicardial Access

Although the conventional subxiphoid approach remains the standard technique for epicardial access, several refinements have been proposed to improve procedural control and safety. One notable modification is the “needle-in-needle” technique, in which a fine micropuncture needle is advanced through a larger introducer needle in a stepwise manner [12]. This controlled approach allows more precise manipulation of the needle tip and reduces the likelihood of inadvertent myocardial puncture. Early clinical experience suggests that this method can facilitate safe access even in anatomically complex situations. In addition, a new technique for percutaneous epicardial access using a curved guidewire has been reported [13]. Another important development is the integration of three-dimensional electroanatomic mapping to guide pericardial access [14]. In this approach, the needle tip can be visualized in real time within the mapping system, and progressive changes in unipolar electrogram amplitude provide feedback as the needle traverses the mediastinum, contacts the pericardium, and enters the pericardial space. Experimental and clinical studies have shown successful access without major complications and with progressively shorter fluoroscopy times, including cases achieved with minimal or no fluoroscopic guidance. This technique may be particularly useful in challenging anatomical contexts, such as in patients with prior cardiac surgery or suspected pericardial adhesions.

### 2.3 Complications Related to Epicardial Access

Percutaneous epicardial access can lead to several serious complications. The commonly used posterior approach may cause injury or bleeding in abdominal organs due to an accidental diaphragm puncture. Liver injury, bowel injury, and intra-abdominal bleeding have been reported, and some patients may have abdominal pain or rebound tenderness [15]. In the anterior approach, there is a risk of damaging the superior epigastric artery or the left internal mammary artery (LIMA), but the complication rate is lower than that of the posterior approach [15]. In one retrospective study of 211 patients, complications occurred in 4.9% with the anterior approach and 10.1% with the posterior approach, supporting the safety of the anterior approach method. A report by Fukuzawa *et al*. [16] showed on CT that the drainage tube path avoided the diaphragm and abdominal cavity after an anterior pericardial access. This confirmed the anatomical safety and feasibility of the anterior approach.

The most common complication is pericarditis, with some reports showing an incidence as high as 30% [17]. In mild cases, only chest pain or ST changes are seen, but in rare instances, it may progress to constrictive pericarditis. Treatment includes NSAIDs, colchicine, or intrapericardial steroid injections [18]. A multicenter prospective study showed that combining intrapericardial steroids with peri-procedural colchicine reduced the rate of pericarditis to 3.1%, with a significant difference compared to intrapericardial steroid injection alone (13.2% → 3.1%, *p* = 0.030). It also significantly reduced the pericardial pain (10.9% vs. 30.9%, *p* = 0.001), pericardial ECG changes (5.4% vs. 33.8%, *p* < 0.001), and new-onset atrial fibrillation (0.8% vs. 19.5%, *p* < 0.001) [19]. Cardiac tamponade and bloody pericardial effusion can be caused by an RV perforation by the needle or guidewire, injury to pericardial vessels, or tearing during sheath manipulation in cases with adhesions. Early bleeding usually occurs within 10 minutes of the procedure and is often due to a right ventricle puncture or vessel injury. Immediate evaluation by transthoracic or intracardiac echo is helpful. Most cases can be managed with drainage and conservative care, but coronary bleeding may require an endovascular or surgical repair. Other rare complications include coronary artery injury due to ablation, phrenic nerve damage, lung or pleural injury, and esophageal injury. Coronary artery injury from ablation tends to occur when the distance is less than 5 mm [20], so coronary angiography or CT should be done before the procedure. For phrenic nerve injury, identifying the capture site by high-output pacing and physically moving the nerve using a balloon [21] or CO_2_ injection is effective. As shown above, epicardial access involves many possible complications. Careful imaging before the procedure, a skilled technique, and close monitoring after the operation are all very important.

## 3. Pathophysiology of Ventricular Tachycardia and Epicardial Ablation Based on Structural Heart Disease

In patients with structural heart disease, VT is most commonly caused by reentrant circuits, which are closely associated with myocardial scarring and conduction abnormalities. Within areas of scar, surviving bundles of myocardial tissue may exhibit slow conduction, giving rise to reentrant pathways. These narrow conduction channels, known as the VT isthmus, play a central role in sustaining the arrhythmia. The VT isthmus is formed by structural electrical barriers caused by surrounding fibrosis and anisotropic myocardial conduction. It is typically bounded on both sides by fixed lines of block (LOBs) and often displays a U-shaped boundary when viewed from above [22]. These LOB-surrounded zones represent the primary targets for VT ablation, and accurate localization requires a careful analysis of local electrograms in combination with advanced mapping techniques. During sinus rhythm, late potentials (LPs) are typically observed between the terminal portion of the QRS complex and early diastole, while during VT, they appear as mid-diastolic potentials (MDPs). When the critical isthmus or lines of block are located in the epicardium, mapping and ablation become more challenging. In these cases, abnormal potentials may only be detected from the epicardial surface, and endocardial mapping may fail to reveal the critical substrate. Epicardial conduction channels are often associated with epicardial scar tissue, which makes ablation technically more complex and sometimes less effective.

### 3.1 Differences in Ventricular Tachycardia Substrate Distribution by Underlying Structural Heart Disease

The distribution of VT substrates differs significantly between ICM and NICM. Therefore, the possibility of requiring epicardial ablation should be anticipated according to the underlying structural heart disease.

#### 3.1.1 Ventricular Tachycardia in Ischemic Cardiomyopathy

In ICM, scar tissue is typically located in the subendocardial layer and often corresponds to areas of prior myocardial infarction. This anatomical correlation allows for relatively predictable identification of VT circuits, and endocardial ablation is generally attempted as the initial approach. However, in some cases, VT circuits may be located in the epicardial or mid-myocardial layers. The need for an epicardial approach should be assessed by integrating findings from mapping, electrocardiography, and cardiac imaging. In particular, for monomorphic VT originating from an inferior scar, an epicardial approach should be considered when the QRS axis exhibits superior or negative concordance across the precordial leads [23]. An epicardial approach should also be considered in cases of recurrence after the first procedure, in patients with large ventricular aneurysms, or when preprocedural VT clearly originates from the epicardium. In such cases, ECG findings can provide important clues. Reported markers include an initial Q wave in lead I, Q waves in leads II, III, and aVF, and an aVR/aVL ratio <1, as well as delayed QRS onset indices such as a pseudo-delta wave ≥34 ms, intrinsicoid deflection time ≥85 ms in lead V2, and a maximum deflection index ≥0.55. These features help to identify patients who are more likely to have an epicardial substrate.

#### 3.1.2 Ventricular Tachycardia in Nonischemic Cardiomyopathy

In NICM, the scar distribution is more heterogeneous and often involves the mid-myocardium and epicardium, making an epicardial approach frequently necessary. Scar tissue in NICM tends to form complex three-dimensional patterns, which makes substrate identification and mapping particularly challenging. It is also common for multiple VT circuits to coexist, and in many cases, the location of the isthmus cannot be clearly defined. According to the HELP-VT study, VT ablation in NICM achieved an acute success rate of 66.7%, which was comparable to 77.4% in ICM (*p* = 0.125), but the long-term outcomes were significantly worse, with a 1-year VT-free survival rate of 40.5% versus 57.0% (*p* = 0.039) [2]. According to a study by Vaseghi *et al*. [24], among 780 patients with NICM (mean age 57 ± 14 years, 18% female, left ventricular ejection fraction 37 ± 13%), the prevalence of underlying heart disease was as follows: idiopathic dilated cardiomyopathy (DCM) 66%, arrhythmogenic right ventricular cardiomyopathy (ARVC) 13%, valvular cardiomyopathy 6%, myocarditis 6%, hypertrophic cardiomyopathy (HCM) 4%, and cardiac sarcoidosis 3%. The 1-year freedom from VT was 69%, and the event-free survival rate for VT, cardiac transplantation, or death was 62%. The 1-year VT-free survival was higher in ARVC (82%) compared with DCM (68%) (*p* ≤ 0.01), as reported in the registry. Valvular cardiomyopathy showed the lowest 1-year VT-free survival rate at 47% (*p* < 0.01), followed by sarcoidosis at 50% and HCM at 55%. The risk of VT recurrence was highest in patients with HCM, valvular cardiomyopathy, and sarcoidosis [24].

#### 3.1.3 Ventricular Tachycardia in Idiopathic Dilated Cardiomyopathy

The most critical step in formulating a VT ablation strategy for DCM is to classify patients into either an antero-septal or infero-lateral pattern based on scar distribution identified by cardiac MRI. The prevalence of antero-septal and infero-lateral patterns is approximately equal. Antero-septal patterns are more commonly associated with endocardial VT, whereas infero-lateral patterns are often linked to VT requiring an epicardial approach [25]. Therefore, an epicardial approach is generally essential in patients with an infero-lateral pattern of DCM. In patients with DCM, LPs were detected in 11% of antero-septal patterns on the endocardium, and in 81% of infero-lateral patterns on the epicardium. These data suggest that abnormal electrograms serving as ablation targets are much more commonly found on the epicardium in the infero-lateral pattern [25]. Consequently, VT ablation outcomes are significantly worse for the antero-septal pattern than the infero-lateral pattern. The antero-septal pattern is particularly challenging, often requiring additional ablation techniques such as simultaneous unipolar radiofrequency (SURF) ablation, half-normal saline irrigation, bipolar ablation, needle ablation, or ethanol injections [26]. For further details on bipolar ablation, please refer to section 6.

#### 3.1.4 Ventricular Tachycardia in Hypertrophic Cardiomyopathy

In patients with HCM, fibrotic areas are frequently observed in the mid-myocardium and epicardium [27]. LV fibrosis often involves both endocardial and epicardial substrates, and VT circuits in HCM have been shown to be localized within intramural and epicardial regions. Santangeli *et al*. [28] demonstrated moderate efficacy of VT ablation in HCM patients with drug-refractory ventricular tachycardia. The exit sites of VT circuits were most commonly located at the junction between the left and right ventricles, either at the basal or apical level. They reported that epicardial ablation was required for VT treatment in approximately 60% of cases [28]. Dukkipati *et al*. [29] reported that a combined endocardial and epicardial mapping and ablation strategy was effective in a selected group of patients with monomorphic VT associated with HCM. In HCM patients with apical aneurysms, low-voltage areas and LPs are often found within the aneurysm, and these frequently serve as targets for catheter ablation [30]. In areas where wall thinning occurs within the apical aneurysm, endocardial ablation may be feasible. However, epicardial access may be required in selected cases, particularly when the arrhythmogenic substrate extends beyond the endocardial layer or when catheter control is limited. In cases where a mid-ventricular obstruction or narrowing is present, catheter control within the apical aneurysm can be technically challenging.

#### 3.1.5 Ventricular Tachycardia in Arrhythmogenic Right Ventricular Cardiomyopathy

In ARVC, fibrofatty degeneration typically progresses from the epicardium toward the endocardium [31], and LPs and fractionated electrograms are often widely distributed over the epicardial surface. As a result, epicardial ablation is often required for effective VT ablation in patients with ARVC.

The ablation targets are typically located in the basal region around the tricuspid annulus and right ventricular outflow tract (RVOT), often extending to the anterior and inferior walls of the right ventricle [32]. Clearly visible scar formation in the RV apex is uncommon, and this region is rarely targeted for ablation. Preprocedural cardiac MRI is useful for assessing the distribution of late gadolinium enhancement (LGE). It is common practice to determine the need for epicardial access based on the findings of endocardial mapping. If low-voltage areas are limited on the endocardium, it is recommended to consider an epicardial approach from the first ablation procedure. On the other hand, when the endocardial substrate is extensive, it is usually better to start with endocardial ablation and add epicardial ablation only if necessary. For patients with monomorphic VT in ARVC, initial catheter ablation has been shown to significantly reduce clinical events, including VT recurrence, cardiovascular hospitalization, and mortality [33].

#### 3.1.6 Ventricular Tachycardia in Cardiac Sarcoidosis

VT episodes associated with cardiac sarcoidosis can be life-threatening and are often associated with a poor prognosis, as the disease is progressive and difficult to treat due to its patchy and extensive epicardial and septal substrates.

In patients with cardiac sarcoidosis who underwent multiple ablation procedures, the 1-year VT-free survival rate was 37%, indicating a poor outcome [34]. Corticosteroid therapy is the primary treatment for cardiac sarcoidosis. In many patients, the first VT episode occurs soon after the initiation of corticosteroid therapy, presumably because of inflammatory conditions, as suggested by Segawa *et al*. [35]. VT storms tend to occur in a bimodal pattern, with peaks in the early and very late phases of the disease, and relatively few events in between. A positive gallium scan is strongly associated with VT, suggesting that inflammation contributes to arrhythmogenesis and that corticosteroid therapy may suppress VT by reducing inflammation [35]. VT associated with cardiac sarcoidosis is frequently accompanied by LPs, and an epicardial approach is often required based on clinical experience.

#### 3.1.7 Ventricular Tachycardia in Post-myocarditis

Myocarditis is one of the most common causes of scar formation in the lateral wall of the LV in young patients [36]. Myocarditis is often suspected in patients who present with symptoms similar to those of acute coronary syndrome but have no significant coronary artery abnormalities. Since the ECG and echocardiography usually provide non-specific findings, cardiac MRI is useful for demonstrating myocardial inflammation and scar in suspected myocarditis [37]. In the acute phase, myocardial edema, subepicardial or sometimes transmural LGE, and regional wall thickening may be observed. As inflammation resolves, the residual scar typically remains on the epicardial layer. The inferolateral wall is the typical site of involvement. In VT associated with myocarditis, numerous LPs are often recorded on the epicardium, and epicardial ablation has shown favorable outcomes [38]. Septal involvement has been reported more frequently in SARS-CoV-2–related myocarditis, in contrast to the typical inferolateral distribution seen in other forms of myocarditis [39].

#### 3.1.8 Ventricular Tachycardia in Valvular Cardiomyopathy

Catheter ablation of VT after valvular surgery generally shows limited effectiveness [24]. This is often due to technical challenges related to postoperative scarring and limited access to the LV. In a study by Liang *et al*. [40] on VT ablation after aortic valve replacement, periaortic scarring was observed in all cases and was involved in the clinical VT circuit in 34% of patients. However, 59% of the VT substrates were unrelated to the valve replacement itself [40]. Approaches to access the LV included transaortic and transseptal routes, which were associated with high procedural safety. In this cohort, ablation was mainly performed endocardially, and epicardial access was rarely attempted because of prior surgery [40]. Soejima *et al*. [41] demonstrated that epicardial or direct surgical ablation was effective in patients with mechanical valves, where LV access was not possible.

## 4. Conventional and Previously Reported Mapping Techniques for Ventricular Tachycardia

A thorough understanding of mapping strategies is essential when considering an epicardial approach for VT. Conventional functional mapping consists of activation, entrainment, and pace mapping [42]. Activation mapping can define the VT isthmus by displaying the site of earliest activation, while entrainment identifies the relation of a pacing site to the tachycardia circuit through features such as concealed fusion and the post-pacing interval. These methods provide precise localization but require VT to be inducible and hemodynamically stable, which is often not feasible in patients with unstable epicardial VT or in cases with multiple morphologies. Pace mapping compares QRS morphologies from pacing with those of the clinical VT and is useful when VT cannot be induced or sustained. It has been enhanced by computer algorithms such as PASO (CARTO™, Biosense Webster, Diamond Bar, CA, USA) and Score (EnSite™, Abbott, Abbott Park, IL, USA). However, accuracy is reduced when multiple circuits exist or epicardial breakthroughs complicate propagation. Thus, conventional mapping remains valuable but is now mainly supportive and interpreted in conjunction with substrate-based mapping.

Substrate-based mapping has become the cornerstone for epicardial ablation because it allows the identification of abnormal electrograms during sinus rhythm or pacing. Low-voltage areas (<0.5 mV), LPs, and local abnormal ventricular activities (LAVAs) are typical markers of arrhythmogenic substrate, and their elimination, including from the epicardium, is associated with improved outcomes [43, 44, 45]. Substrate-guided approaches such as scar homogenization, dechanneling, and core isolation have been proposed, although the targeted ablation area can differ depending on operator and patient characteristics [46, 47, 48]. To overcome the limitations of voltage mapping alone, several functional refinements have been introduced.

DEEP mapping was first described by Jackson *et al*. [49], who showed that LPs with a conduction delay of ≥10 ms after an extrastimulus were more specific for critical isthmus sites than ordinary LPs. Their mapping protocol involved RV pacing at a cycle length of 600 ms, with a single extrastimulus delivered at a coupling interval of the ventricular effective refractory period plus 20 ms each time an LP or fractionated potential was identified. In their small series of ischemic VT, DEEP sites colocalized with diastolic pathways and improved specificity without loss of sensitivity. Porta-Sánchez *et al*. [50] later validated this in 20 patients with ICM, showing that DEEPs comprised a smaller proportion of abnormal signals (4.8% vs. 16.8% for LPs) but overlapped more closely with the VT isthmus, and ablation at DEEP sites led to 75% VT-free survival at 6 months.

EDP mapping, introduced by de Riva *et al*. [51], further tested this concept by analyzing 60 patients with ICM-VT. EDPs were defined as >10 ms conduction delay or block after an extrastimulus. They were observed in 62% of cases and often localized to regions that appeared normal on bipolar voltage mapping but matched areas of LGE on MRI. Ablation guided by EDPs was associated with reduced recurrence. These results highlight how functional stimulation can reveal hidden substrate, especially in patients with small scars or epicardial involvement, where voltage mapping underestimates abnormal myocardium.

Isochronal late activation mapping (ILAM), described by Irie *et al*. [52], uses local activation time maps during sinus rhythm or pacing. Isochrones are divided into eight intervals, and the spacing reflects conduction velocity. Narrow spacing marks slow conduction, and a “deceleration zone” (DZ) is defined when three or more isochrones crowd within 1 cm. Subsequent studies, including a large series by Aziz *et al*. [42], showed that DZs overlapped with the VT isthmus in most cases, and ablation targeting DZs yielded high acute success and VT-free survival (80% in ICM, 63% in NICM). Importantly, ILAM allows systematic visualization of conduction slowing even when VT is not inducible, making it highly relevant in epicardial mapping.

Rotational activation patterns (RAPs), reported by Hattori *et al*. [53], represent another functional refinement. RAPs are defined by a >90° bend of the activation wavefront occurring at the border or within a slow conduction zone. In their study of 45 VTs, RAPs were present in 70% of critical isthmus regions and became more evident when the wavefront direction was altered by pacing. RAPs may be absent in purely intramural or epicardial circuits, but when present, they provide a direct marker for critical sites.

Together, these refinements—DEEP, EDP, ILAM, and RAP analysis—extend the diagnostic value of substrate mapping. They are especially useful in epicardial VT, where conventional mapping is limited, and allow operators to better identify and target critical substrates for ablation.

## 5. Advancements and Expert Tips of Substrate Mapping

In challenging cases that require epicardial ablation, the mapping techniques described in Section 3 are often insufficient in practice. This section outlines the mapping techniques used in our center for difficult cases, described separately for the CARTO™ and EnSite™ systems.

### 5.1 Visualization of Electrophysiologic Abnormalities Using the CARTO™ System

#### 5.1.1 Ripple Mapping

Ripple mapping in the CARTO™ system displays electrical activation as moving bars based on recorded electrograms. The height of each bar reflects the local bipolar voltage and rises or falls as time progresses. When electrograms are recorded densely and uniformly across the entire map, the bars move from one area to another, creating a ripple-like effect that shows how the activation wavefront spreads. Ripple mapping is the only algorithm that simultaneously displays both the voltage and activation by tracking the sequence of bar movements, allowing visualization of the local conduction across the entire map. VT circuits are associated with delayed conduction through surviving myocardial fibers within scar tissue, typically appearing as fragmented low-amplitude signals in the late phase of sinus rhythm. Standard annotation methods in three-dimensional (3D) mapping may not distinguish delayed local potentials within scar from initial far-field signals coming from the surrounding healthy tissue, but ripple mapping may help overcome this limitation. In VT isthmus regions, ripple mapping shows multiple small ripples that move slowly and represent fragmented signals, whereas normal myocardium displays a single tall bar reflecting simple signals. Luther *et al*. [54] reported that ripple mapping using CARTO™ can identify conduction channels within scar, enabling a functional substrate ablation. Katritsis *et al*. [55] published a multicenter prospective study evaluating VT substrate ablation by targeting scar channels to eliminate LPs without direct ablation. They concluded that scar channel ablation is feasible by ripple mapping and can be an alternative to more extensive substrate modification techniques [55]. Ripple mapping can suggest the presence of epicardial VT circuits during endocardial mapping.

#### 5.1.2 Early Meets Late Lower Threshold

**Fig. 2.**
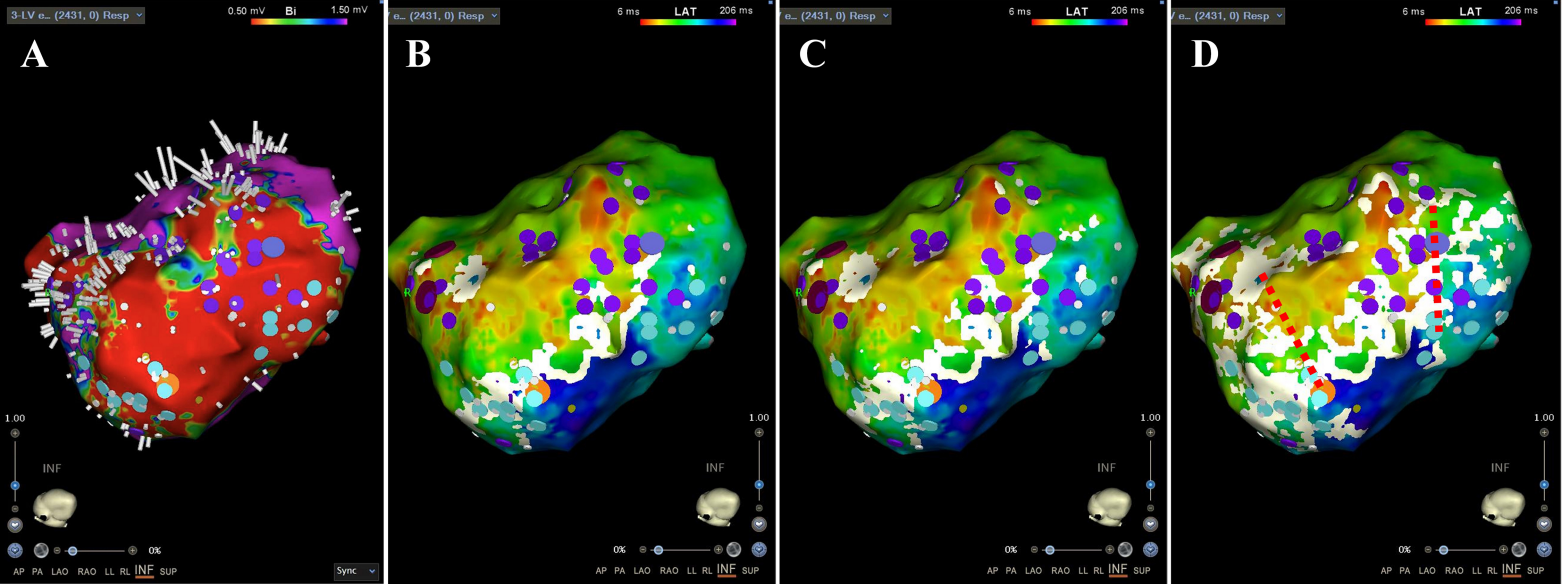
**Relationship between the EML lower threshold white lines and VT isthmus on the substrate map**. This is a case of VT due to an old inferior myocardial infarction. (A) The substrate map during sinus rhythm exhibited a large low-voltage zone on the inferior wall. The ripple mapping video is provided as Video 2. (B) The map created during sinus rhythm shows the EML lower threshold set at 30. The LOB is shown as a white line. (C) The EML lower threshold has been changed to 20. (D) The EML lower threshold has been changed to 15. When the EML threshold is lowered, “incomplete LOBs” appear. These incomplete LOBs may include fractionation potentials, abnormal signals (LPs and LAVAs), and the VT isthmus. By looking at the new white lines created by lowering the threshold, the VT isthmus (red dashed line) can be estimated. Light blue tags show LPs, and purple tags LAVAs. EML, early meets late; VT, ventricular tachycardia; LOB, line of block; LP, late potential; LAVA, local abnormal ventricular activities.

A substrate map is created using the CARTO3™ system during sinus rhythm or high right atrial pacing (Fig. 2). The “early meets late (EML) lower threshold” function is used to identify regions with suspected abnormal electrograms, based on the following steps:

(1) Total Activation Time: Calculate the time difference between the earliest and latest activation points on the map.

(2) Calculate the local activation time difference between each pair of neighboring points.

(3) Express each local activation time difference as a percentage of the total activation time, and draw a white line between points that exceed the threshold.

**Video 2.** **Ripple mapping of the VT substrate in the case presented in Fig. 2**. Video associated with this article can be found in the online version at https://doi.org/10.31083/RCM44332.

The EML lower threshold function is typically used to highlight areas where there is a significant delay in the activation between two points, such as in double potentials. However, for detecting local abnormal signals such as LPs and LAVAs, lowering the threshold to around 10–20% improves the diagnostic sensitivity. Using a higher EML lower threshold improves the specificity, while lowering it enhances the sensitivity for detecting LPs and LAVAs. However, the optimal EML lower threshold varies depending on the characteristics of abnormal potentials within the VT isthmus. At our institution, the EML lower threshold is used in combination with ripple mapping to help identify complex VT circuits. This approach is useful for complex VT ablation of both endocardial and epicardial origins.

#### 5.1.3 Multipolar Mapping

**Fig. 3.**
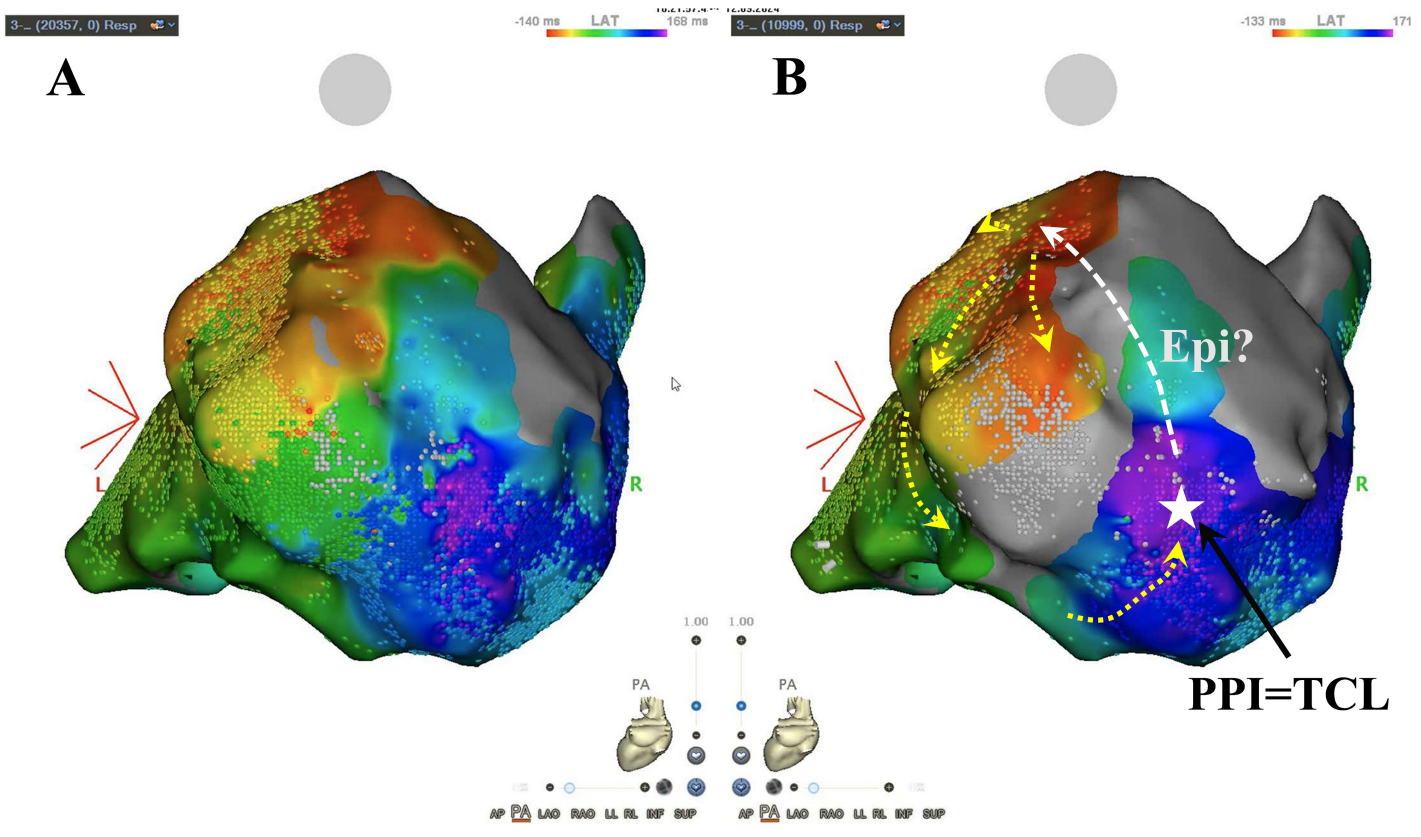
**Bipolar LAT mapping with wavefront annotation and multipolar mapping**. This is a case of VT with cardiac sarcoidosis. Substrate mapping was performed from the left ventricular endocardium. (A) shows bipolar LAT mapping with wavefront annotation. (B) shows multipolar mapping in the same case. In the bipolar LAT mapping with wavefront annotation, the activation appears to spread continuously, but in the multipolar mapping, conduction stops at the posterior wall and converges to the anterior wall, suggesting partial epicardial conduction. On the endocardial side, the PPI matched the TCL, and ablation at that site was successful. The ripple mapping video is provided as Video 3. The ripple mapping video is provided as Video 3, demonstrating ripple mapping during wavefront annotation and multipolar mapping in this case. The yellow dashed line indicates the direction of endocardial activation, whereas the white dashed line represents the presumed epicardial conduction pathway. LAT, local activation time; PPI, post-pacing interval; TCL, tachycardia cycle length.

Multipolar mapping is a novel technique that records local electrograms regardless of the activation direction, unlike conventional bipolar or unipolar methods (Fig. 3). It is available in the CARTO3™ system when mapping is performed with the OPTRELL™ catheter. The system removes far-field components by comparing each unipolar signal from the OPTRELL™ electrodes with those from the eight surrounding electrodes, allowing clearer identification of local potentials. Subtracting far-field signals shared with nearby electrodes allows each electrode to produce a multipolar signal that highlights its local component. Multipolar signals are unipolar electrograms adjusted to reduce far-field interference, and annotations are placed at the point of the maximum slope (max–dV/dt) of each signal. In various arrhythmia cases, including VT, multipolar mapping has been reported to work better than conventional methods by more effectively removing far-field components, preserving local signals, improving voltage accuracy, and helping to identify the arrhythmia origin [56]. When used for endocardial VT activation mapping, this method can suggest epicardial conduction on the map, helping to determine whether an epicardial approach should be considered.

**Video 3.** **Ripple mapping during wavefront annotation and multipolar mapping (Fig. 3)**. Video associated with this article can be found in the online version at https://doi.org/10.31083/RCM44332.

#### 5.1.4 Complex Signal Identification

The Complex signal identification (CSI) function, available in CARTO3™ version 8, is an algorithm designed to automatically detect complex potentials in the atrium and mark their locations on the map. It was originally developed to identify fractionated potentials during atrial flutter (AFL), using a machine learning model trained on data labeled by experienced physicians. CSI analyzes bipolar signals and assigns a fractionation score ranging from 0 to 10 at each point. The score is calculated based on parameters such as the local potential duration, amplitude, and activation timing. Higher scores indicate greater specificity in detecting fractionation but may reduce sensitivity. Although initially intended for AFL mapping, CSI is now also used to identify fractionated potentials during sinus rhythm or atrial pacing, and its application has expanded to other atrial arrhythmias such as atrial fibrillation (AF) [57]. Fractionated potentials are also known to exist within ventricular scar tissue associated with VT, suggesting that CSI may be useful for automatic visualization of these signals in the ventricle. In VT ablation, electrogram and electroanatomical mapping have traditionally been used to identify scar areas and slow conduction pathways. However, detecting abnormal signals and clearly defining circuit structures can be difficult in patients with extensive and complex scarring. As a result, there is growing interest in applying CSI-based automated detection of fractionated potentials—originally developed for atrial arrhythmias—to ventricular mapping. Although CSI was originally designed for atrial mapping and has not yet been fully tested in the ventricles, future improvements in the algorithm and wider application may make it a useful tool for VT ablation.

### 5.2 Visualization of Electrophysiologic Abnormalities Using the EnSite™ System

#### 5.2.1 Visualization of QRS Fractionation Using the Fractionation Map

We use the fractionation map in the EnSite™ system as a tool to show QRS fractionation potentials when checking VT substrates. This function was first made to find complex fractionated atrial electrograms in atrial arrhythmias. The fractionation map can find small waves (fractionated components) in local electrograms and count them. To do this analysis, we must set the values for the sensitivity, width, and refractory. Based on those settings, small waveforms (like sine curves) that match the rules are seen as one “component”. Each detected component gets a marker on the electrocardiogram. The total number of markers is displayed as the fractionation number for the point where the local electrogram was recorded. By setting a fractionation threshold, only points with fractionation numbers above the threshold are highlighted on the map with yellow dots. The fractionation map makes it easy to see which areas have high fractionation of the electrogram. The minimum settings are 5 ms for the width and 6 ms for the refractory period, which may result in very small fractionated potentials below these thresholds not being displayed on the map. This is the only weakness of this method. Using the fractionation map during sinus rhythm or pacing helps identify abnormal electrograms and slow conduction zones that may not be visible with conventional mapping. It can also be applied to epicardial mapping and may be useful as an additional method for assessing VT substrates.

#### 5.2.2 EnSite OT Near Field™ (OTNF™) Algorithm

In conventional practice, the shape of the local electrogram waveform has been used to judge the characteristics of the signal. In general, sharp waveforms are thought to indicate near-field signals and usually contain higher frequency components. In contrast, dull waveforms are typically far-field signals and mainly contain lower frequency components. In scarred myocardium, electrograms often include both near-field and far-field components, which makes the signals complex. To detect high-frequency components within these complex signals, the EnSite™ X system uses a specialized algorithm called EnSite OT Near Field™ (OTNF™). This algorithm automatically identifies the highest frequency component—called the peak frequency (PF)—from recorded bipolar or omnipolar signals and adds an annotation at that point [58]. The PF is a number that shows how sharp the waveform is. It is calculated using a wavelet analysis. The wavelet analysis divides the signal into short wavelets, allowing identification of when and where specific frequencies appear over time. The results are shown as a PF trace, a line that tracks how the frequency changes over time.

From the PF trace, two key pieces of information can be obtained at each site where a local electrogram is recorded:

1. PF_max_: the highest frequency value recorded at that site.

2. Local activation time: the timing when the PF_max_ appears.

The wavelet-based PF used in OTNF™ has distinct characteristics compared to conventional frequency analyses using the Fourier transform (FFT). The FFT changes the signal into continuous sine and cosine waves, which results in a loss of time information. As a result, the FFT tends to emphasize the dominant frequency—the one with the highest energy. As a result, the FFT mainly highlights the dominant frequency with the highest energy calculated as the square of the amplitude. In contrast, OTNF™ can detect high-frequency components even with low energy, while preserving time information. To avoid mistaking small noise for true high-frequency signals, OTNF™ only analyzes waveforms with peak-to-peak voltages of 0.04 mV or higher. In OTNF™-based measurements, a 60-ms window (30 ms before and after the PF annotation) is analyzed, and the highest voltage within this range is used as the local signal amplitude. In summary, the OTNF™ algorithm is a novel tool that efficiently detects high-frequency components—characteristic of near-field signals—within complex electrograms and may be clinically useful for identifying abnormal conduction pathways or selecting appropriate ablation targets, especially in scarred tissue.

#### 5.2.3 Challenges in Identifying 3D VT Circuits and the Role of OTNF™

VT circuits often have complex 3D structures. Current contact mapping techniques can only record signals from surface areas such as the endocardium or epicardium, making it difficult to evaluate intramural circuits. Previous studies using sequential and simultaneous endo- and epicardial mapping (SEEM) have shown that most VT circuits follow three-dimensional pathways across the myocardial wall, and that only 17% remain within a single two-dimensional (2D) plane [59]. SEEM cannot be performed in all cases, so there is a need for easier ways to obtain information about 3D VT circuits. Tonko *et al*. [60] examined whether detecting near-field signals could help solve this problem. Using near-field detection, complete mapping of the diastolic wavefront through the VT isthmus was achieved in only 16.6% of cases, while partial identification was possible in 61.1%. This suggests that near-field mapping works well for VT circuits in a 2D plane, but often fails in more complex 3D VT circuits. Therefore, when near-field mapping is complete, the VT circuit may be superficial and easier to ablate. In contrast, incomplete near-field mapping may suggest a deeper and more challenging circuit, serving as a diagnostic clue. Specifically, if diastolic activation appears continuous and complete without isochronal gaps on a single surface, a 2D VT circuit is suspected. In contrast, if parts of the circuit appear absent or if diastolic potentials are not observed in near-field mapping, a deep 3D VT circuit may be involved. However, this approach has limitations, as poor catheter contact or epicardial fat may make near-field signal detection more difficult.

#### 5.2.4 Peak Frequency Map and Emphasis Map

The OTNF™ algorithm includes two tools: the peak frequency map and the emphasis map. The peak frequency map shows the PF calculated for each electrogram. Areas with low frequencies (such as ≤200 Hz) are shown in gray, and areas with high frequencies (such as ≥250 Hz [61]) are shown in white. Intermediate frequencies between these two ranges are displayed in red. The frequency threshold can be adjusted by the user. The emphasis map combines data from existing maps—such as voltage, local activation time, conduction velocity, PF, and fractionation—and highlights the selected features among them.

#### 5.2.5 Substrate Mapping Using Near-field Detection, Peak Frequency, and the Emphasis Map

A method has been proposed that combines near-field detection and the peak frequency analysis to target low-voltage, high-frequency (LVHF) areas. This helps to tell the difference between critical areas that need ablation and bystander areas that do not. During sinus rhythm, assessing LVHF areas (PF >200 Hz) improves the specificity for identifying VT termination sites, compared to low-voltage areas alone (sensitivity/specificity: LV only 0.90/0.63 vs. LVHF 0.89/0.87) [62]. However, one study reported that using a PF >300 Hz alone could not distinguish the VT isthmus from bystander scar [60]. Thus, while near-field detection and PF mapping can provide helpful additional data, they are limited when used alone. In scar-related VT, targeting only LVHF areas is not always effective, so a comprehensive approach is needed.

#### 5.2.6 Our Institutional Approach 

At our institution, we use emphasis maps—including the voltage map, fractionation map, and peak frequency map—during sinus rhythm or pacing (from the right atrium or right ventricle) to estimate the critical sites that should be targeted for ablation. The analysis window for the fractionation map is set from the onset of the QRS complex to the latest point where LPs are detected. This setting allows count markers to be assigned to fractionation potentials both on the QRS complex and just after it. Detecting fractionation potentials both inside and outside the QRS helps identify delayed conduction zones and abnormal signals around the VT isthmus during sinus rhythm or pacing mapping. By gradually lowering the fractionation threshold (e.g., from 9 to 2), we can visualize the conduction pathway of the delayed area on the map. Fractionation points on the fractionation map include both near- and far-field signals and are useful in epicardial VT. But since standard 2D maps only show signals from the catheter’s contact surface, the full conduction path may not be clearly seen. To improve this, we use the peak frequency map to help detect near-field signals. Since the near-field detection threshold varies by case, we gradually raise the frequency cut-off (e.g., from 200 Hz to 600 Hz in 50 Hz steps) to better visualize the VT isthmus on the map. In our experience, areas highlighted by emphasis maps, including the voltage map, fractionation map, and peak frequency map, often correspond to LPs or LAVAs, allowing effective treatment even in complex VT cases (see Fig. 4A,4B,4C,4D).

**Fig. 4A.**
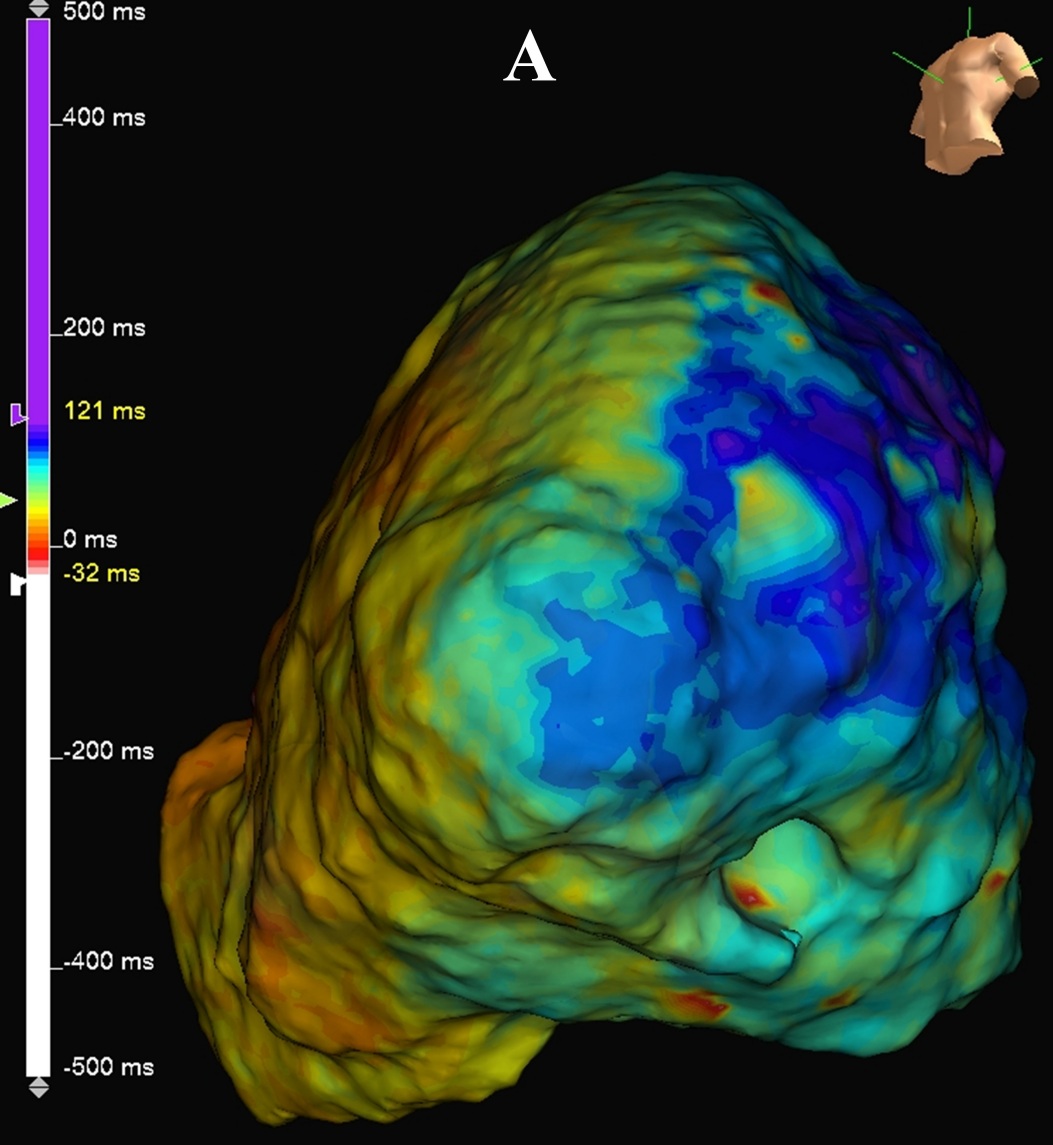
**Complex VT case with hypertrophic cardiomyopathy**. This is a VT case with hypertrophic cardiomyopathy. This shows an ILAM made during right ventricular pacing. DZs are seen at the apex. ILAM, isochronal late activation mapping; DZ, deceleration zone.

**Fig. 4B.**
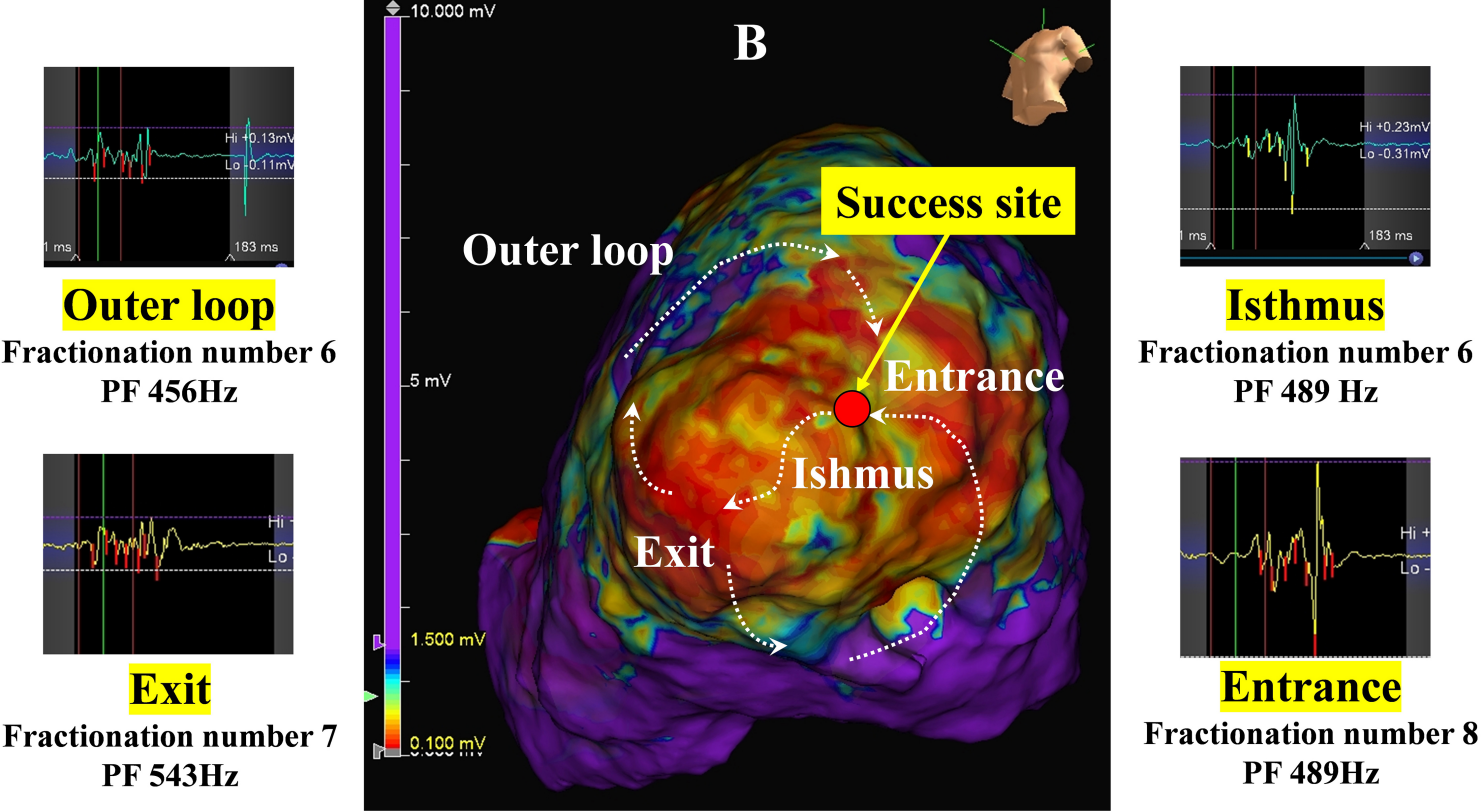
**Local electrocardiograms on and around the VT isthmus**. This is the voltage map made during right ventricular pacing. A low voltage area is seen at the apex. The VT forms a figure-eight circuit around the apex. The estimated circuit and activation direction are shown by the dashed white arrows. The local electrograms, fractionation number, and PF are shown at the entrance, isthmus, exit, and outer loop. Electrograms in the VT circuit have a high fractionation number and PF. Ablation near the entrance was successful. PF, peak frequency.

**Fig. 4C.**
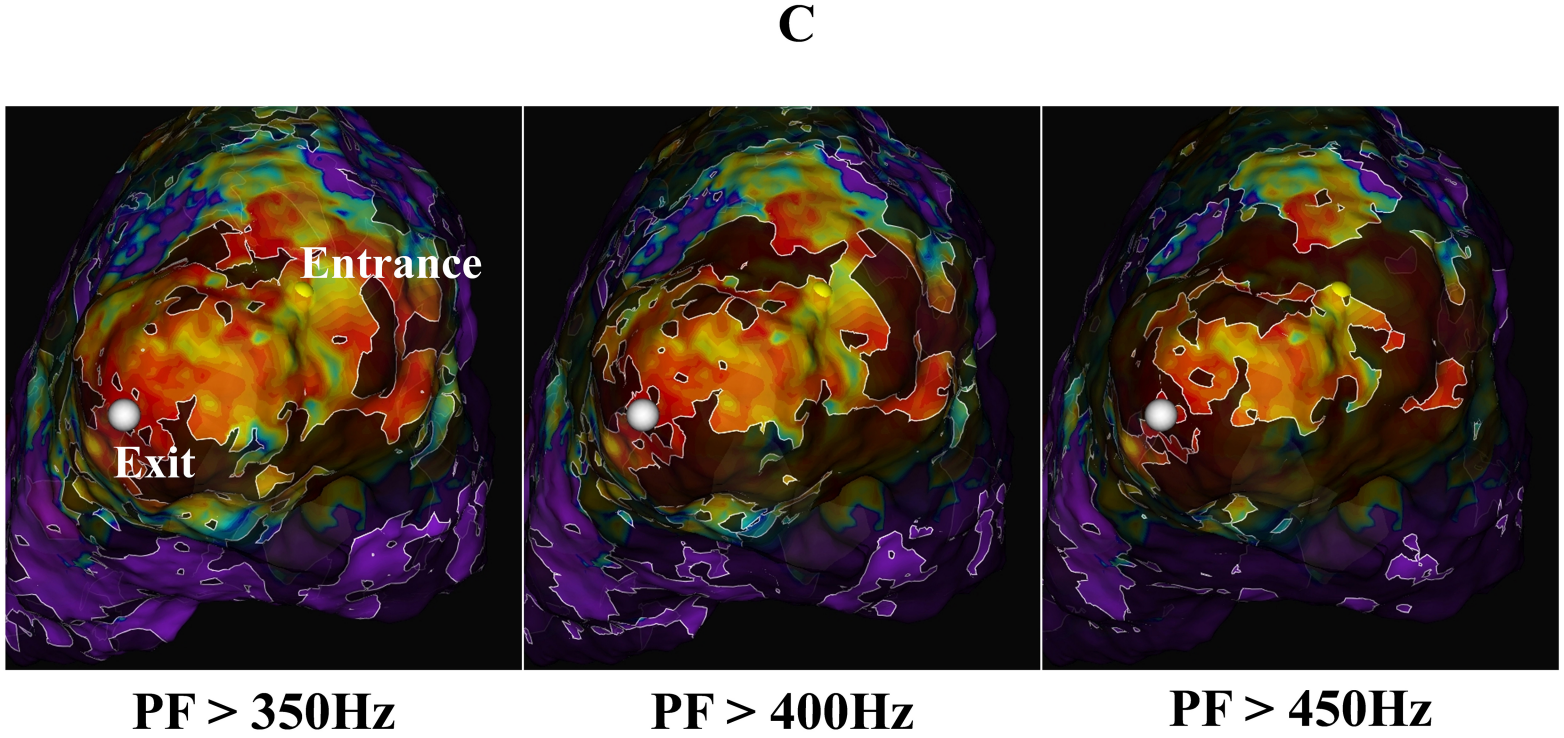
**Emphasis map (voltage map + peak frequency map)**. This is an emphasis map combining the voltage map and peak frequency map. The PF cutoff value was changed step by step by 50 Hz. The highlighted areas show electrograms with a PF above the cutoff. PF cutoff values of 350 Hz, 400 Hz, and 450 Hz are shown. The yellow tags show the VT entrance, and the white tags the VT exit. The VT circuit was visualized at a PF of 400 to 450 Hz. PF, peak frequency.

**Fig. 4D.**
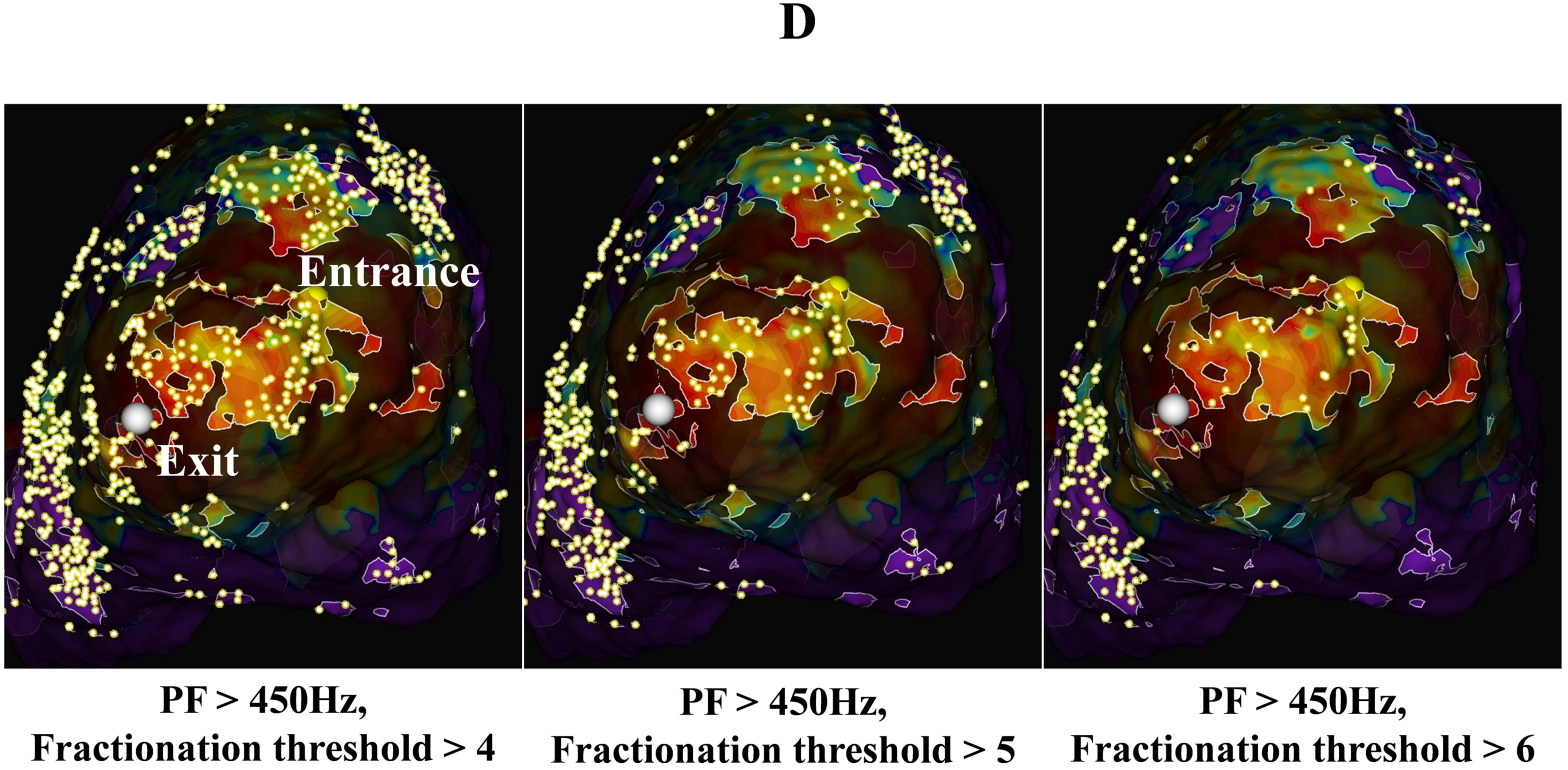
**Emphasis map (voltage map + peak frequency map) + fractionation map**. This is an emphasis map combining the voltage map, PF map, and fractionation map. The PF cutoff was fixed at 450 Hz, and the fractionation threshold was changed. Spot cites, meeting the fractionation threshold, are shown as small fluorescent yellow dots. Fractionation thresholds of 4, 5, and 6 are shown. The VT circuit was seen at thresholds of 5 to 6. PF, peak frequency.

## 6. Challenging Cases and Advanced Strategies

This section explains difficult or complex cases that require epicardial ablation. It focuses mainly on epicardial ablation after prior cardiac surgery and on bipolar ablation.

### 6.1 Epicardial Ventricular Tachycardia Ablation After Open-heart Surgery

#### 6.1.1 Difficulty of Access and the Strategy

**Fig. 5.**
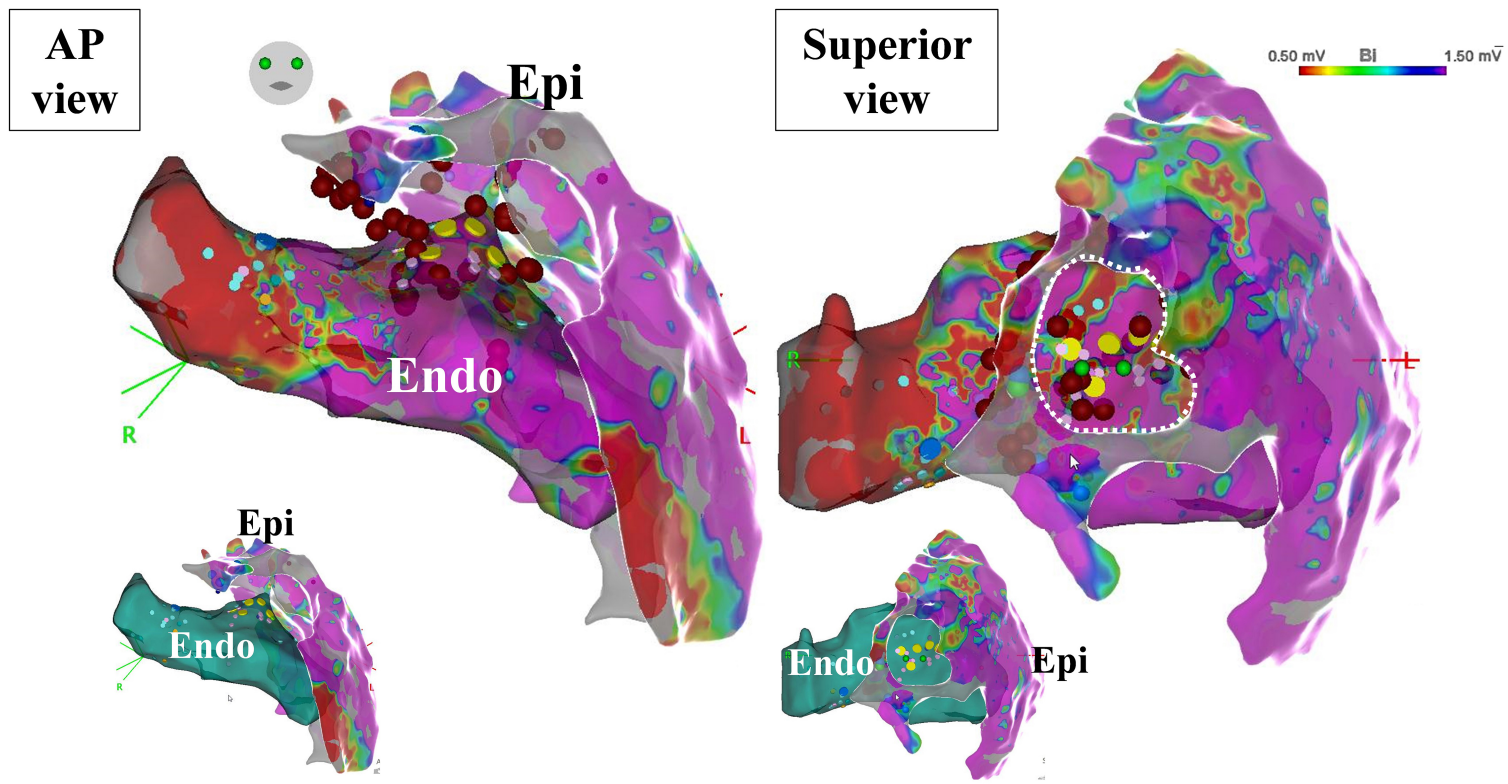
**Epicardial ablation case with prior open-heart surgery**. Epicardial ablation was performed in a patient who had open-heart surgery (CABG and mitral valve repair) 9 years prior. Substrate maps were created from the left ventricular endocardium and epicardium. The small figure shows only a light-blue anatomical model for the endocardium to make the epicardial map easier to see. Due to severe adhesions, the catheter could not reach the upper basal anterior wall. This area appears as a donut-shaped gap (white dashed line in the superior view) in the epicardial map. The VT circuit existed in that area, and successful ablation was achieved from the opposite endocardial side. Red tags show ablation sites, and yellow tags indicate good pace map sites. CABG, coronary artery bypass grafting

Epicardial access becomes much harder after open-heart surgery, like coronary artery bypass grafting (CABG) or valve surgery, because of pericardial adhesions. In these cases, the contrast media does not spread widely around the heart but stays in one area near the bottom because of adhesions. Adhesions can also make it hard to move the guidewire freely around the heart. We usually use the anterior approach under the xiphoid first, but if adhesions are severe, we switch to the posterior approach. To break adhesions, we use the ablation catheter or a deflectable sheath (like Agilis) for blunt dissection and try to make a path for the catheter [63]. If access is still difficult, we consider a surgical epicardial access. There are two types of surgical epicardial access: a subxiphoid surgical access and a limited anterior thoracotomy. Both allow VT mapping and ablation without opening the chest [64]. The subxiphoid surgical access uses a vertical cut below the xiphoid. The pericardium is then cut sideways to open the pericardial space. Blunt dissection of adhesions allows safe access to areas like the diaphragmatic surface or inferior wall, which are hard to reach with a needle. The limited anterior thoracotomy uses a small cut on the left front chest (3rd–5th ribs) to enter the chest and expose the front wall of the LV and heart base. The pericardium is opened to remove adhesions on the front wall, allowing epicardial mapping and ablation. During surgery, one-lung ventilation or ECG lead movement may be needed. The method is chosen based on the ablation target area, operator experience, and bypass graft location. In a study by Tschabrunn *et al*. [63], they examined 10 VT patients with past heart surgery or pericarditis. All had adhesions, but 90% could be mapped well using ablation catheters or deflectable sheaths. VT was stopped in 80% of the cases. Complications were minor, and long-term results were good [63]. Killu *et al*. [65] reported that 78% of 18 patients with past heart surgery (including CABG) had a successful percutaneous epicardial access using the posterior approach. Many cases needed an adhesion dissection, but no graft injury occurred. Ablation was successful in 13 patients. However, serious bleeding complications like a pericardial hematoma or tamponade were also reported, showing that this approach, while effective, has some risk [65]. The effect of adhesions after epicardial ablation has also been studied in repeat procedures. In a study by Tschabrunn *et al*. [66], 23% of 30 patients who had a repeat puncture at a median of 110 days after the first procedure showed adhesions. However, epicardial mapping was still possible in 90% of cases, and clinical VT was successfully eliminated [66]. Even when epicardial access is achieved, it may be impossible to reach the expected VT circuit due to severe adhesions. In such cases, as in our experience, ablation from the opposite endocardial side may still be effective (see Fig. 5).

#### 6.1.2 Our Institutional Outcomes

Between 2010 and 2022, we performed VT ablation in 354 patients with sustained VT (421 VTs), and an epicardial approach was considered in 62 patients (18%) for 77 VTs (18%). Five patients (1.4%) with seven VTs (1.7%) had a history of open-heart surgery, and a percutaneous epicardial access was successful in 4 patients (80%) for 6 VTs (86%). Epicardial access failed in only one patient. In two of the successful puncture cases (29%), epicardial ablation was not possible due to adhesions, but both achieved non-inducibility by ablation from the opposite endocardial side. At the end of the procedure, the clinical VT was inducible in only one case, where the epicardial puncture failed. One patient with a successful puncture developed intrapericardial bleeding, which was controlled conservatively with pericardial drainage. No major complications occurred in the other cases. These results suggest that although adhesions after open-heart surgery make epicardial access and ablation more difficult, the epicardial procedure for patients with previous open chest surgery can still be performed safely and effectively with proper planning and strategy, especially in urgent cases or when an epicardial origin is strongly suspected. We also experienced a successful repeat epicardial ablation, showing that re-access is feasible when the degree of adhesions, ablation tools, and approach are carefully selected. In the future, a multimodal strategy including preprocedural imaging for adhesion assessment, individualized puncture routes, and surgical support when needed will be important.

### 6.2 Bipolar Radiofrequency Ablation

#### 6.2.1 Strategic Approach for Refractory VT Involving an Epicardial or Deep Substrate

Bipolar radiofrequency ablation (Bi-RFA) is a promising treatment for ventricular arrhythmias that come from deep areas, the septum, or the LV summit, where standard unipolar ablation (Uni-RFA) is not effective. In Bi-RFA, energy flows between two catheters. This creates deeper lesions by focusing energy at the target site.

#### 6.2.2 Lesion Formation and Basic Data

In animal studies (swine ventricles), Bi-RFA using TactiCath™ at 30 W for 60 seconds with a saline flow of 30 mL/min created transmural lesions in 62% of the tissue with an average thickness of 11.8 mm. The steam pop rate was low, at 3.5%. In comparison, Uni-RFA made transmural lesions in only 7% of thinner tissue (average 4.2 mm), and the steam pop rate was higher at 10%. Bi-RFA was more likely to create full-thickness lesions in the ventricular wall and needed only half the time to make lesions of the same size and depth compared to Uni-RFA [67]. Data on Bi-RFA in humans is still limited [68].

#### 6.2.3 Clinical Study Data

Kany and colleagues [69] reported a multicenter observational study using Bi-RFA for drug-resistant VT or ventricular premature complexes (VPCs). They treated 24 patients with 26 VTs. All VT cases (14/14) and 7 of 12 VPC cases (58%) had acute success. In *ex vivo* experiments, combining Bi-RFA and Uni-RFA made lesion volumes about three times larger than Bi-RFA alone (1429 mm^3^ vs. 423 mm^3^). In clinical practice, this combined strategy was used more often in patients without recurrence (92% vs. 36%), showing its effectiveness [69]. In a registry study from 16 centers in Europe, Futyma and colleagues [70] used Bi-RFA in 91 patients with 94 VTs. Complete success was 74%, partial success was 11%, and the VPC burden was reduced by over 80% in 78% of cases [70]. However, serious complications such as coronary artery occlusions, AV block, and arteriovenous fistulae have also been reported. Careful anatomic planning and safety checks are important.

#### 6.2.4 Strategy and Outcomes by Location

The following sections describe approaches for septal VT, the LV summit, and the LV free wall.

6.2.4.1 Septal Ventricular Tachycardia

Della Bella and colleagues [71] performed Bi-RFA in 21 NICM patients and achieved clinical VT non-inducibility in 95% of them (20 out of 21). If the septum was very thin and the distance between the two catheter tips was less than 5 mm, Bi-RFA could not be done safely. This was the only anatomical limitation. VT recurrence was more common in patients with an extra-septal substrate or inflammatory cardiomyopathy. This suggests that treatment should be adjusted based on the anatomy [71].

6.2.4.2 Left Ventricular Summit

The anatomy of the LV summit has a strong effect on treatment outcomes. Yamada and colleagues [72] divided the LV summit into two parts—inferior apical (A-LV summit) and superior basal (B-LV summit)—based on the location of the great cardiac vein (GCV). In the A-LV summit, they achieved a 100% success rate using both inside-the-GCV and epicardial approaches. In contrast, the success rate in the B-LV summit was only 48%. A close distance to the coronary arteries and thick fat were the main limitations [72]. Based on these findings, Enriquez and colleagues [73] reported a multicenter study in which bipolar ablation was performed by placing catheters between the GCV and the endocardium of the Left ventricular outflow tract (LVOT) or Right ventricular outflow tract (RVOT). In 20 cases where Uni-RFA had failed, they achieved acute success in all patients. After 30 months of follow-up, the recurrence rate was only 15%. No major complications occurred, as they confirmed a safe distance (>5 mm) from the coronary arteries using angiography.

6.2.4.3 Left Ventricular Free Wall

Igarashi *et al*. [74] analyzed 18 Bi-RFA cases from 7 hospitals in Japan. The acute success rate was 89%, including 3 cases (17%) from the LV free wall. After 12 months, VT had recurred in 8 patients (44%). Four of the patients with recurrence needed repeat ablation. The others were controlled with medications or an Implantable cardioverter defibrillator (ICD). Complications during the procedure included 2 cases with atrioventricular (AV) block and 1 case with a coronary artery occlusion. Bi-RFA was useful for the acute control of difficult VTs. Although the long-term recurrence rate was relatively high, the overall VT burden was significantly reduced [74].

#### 6.2.5 Our Institutional Outcomes

At our hospital, Bi-RFA was used in 6 out of 436 sustained VT ablation cases (1.4%) performed between 2012 and 2025. Epicardial access was performed in 83% of the 6 Bi-RFA cases. All patients had NICM (DCM, HCM, or cardiac sarcoidosis), and Bi-RFA was done after an average of 1.8 Uni-RFA sessions. The target sites for Bi-RFA were the septum (n = 2), LV summit (n = 2), mitral annulus, and LV free wall. The average settings were 29 W, 302 ± 193 second RF time, and 17 ± 7.7 mm catheter spacing. The acute success rate was 67%. One case each with a cardiac tamponade, AV block, and skin burn was reported, but all were managed conservatively. Long-term follow-up data (over 12 months) were available for five of the six patients who underwent Bi-RFA at our center. VT recurrence occurred in two patients, while three patients remained free from VT recurrence and did not experience ICD shocks during more than 12 months of follow-up. Although the sample size is small, these preliminary results suggest that Bi-RFA can provide sustained arrhythmia control in selected cases. Bi-RFA can also offer strong lesion formation and good acute success in complex areas such as deep substrates, the septum, and the LV summit. However, careful planning is required due to anatomic limitations such as coronary proximity, fat layers, and thin myocardium. The use of pre-procedural imaging, adequate catheter spacing, and a sufficient safety margin may help improve procedural safety and broaden the clinical applicability of Bi-RFA. Further studies with larger cohorts and longer follow-up are needed to clarify its long-term efficacy and clinical role.

## 7. Future Perspectives

Several new ablation approaches are being explored as potential strategies for treating refractory VT. Pulsed field ablation (PFA) has recently drawn considerable attention as a non-thermal energy source that can create deep, tissue-selective lesions while minimizing the risk of injury to adjacent structures such as the coronary arteries and the phrenic nerve [75, 76]. Early clinical and preclinical studies have shown that PFA is feasible for targeting substrates that are difficult to ablate with traditional radiofrequency energy. Another promising method is coronary venous ethanol ablation. This technique achieves transmural lesion formation by injecting ethanol into selected coronary venous branches and may be particularly useful for intramural substrates or areas that are otherwise unreachable with standard approaches [77, 78].

Beyond the evolution of ablation energy itself, recent advances in artificial intelligence (AI) and computational modeling hold the potential to further reshape strategies for epicardial VT ablation. AI-assisted VT localization systems that integrate multimodal imaging data have shown encouraging results in improving the accuracy of substrate identification and circuit delineation through machine-learning algorithms [79]. Furthermore, the emerging concept of a “digital twin”—a patient-specific heart model built before the procedure—enables virtual simulation of VT circuits and identification of critical regions such as the isthmus [80]. This approach can predict the distribution of arrhythmogenic substrates and the likely reentrant pathways in advance, thereby improving the precision of ablation planning. Although these technologies are still in the early stages of clinical translation, they have the potential to complement and expand existing epicardial ablation strategies, offering new therapeutic options for managing complex ventricular arrhythmias.

## 8. Conclusion

Epicardial ablation has become an essential option in the treatment of VT. Its success depends not only on technical proficiency but also on a deep understanding of the underlying substrate and careful pre-procedural planning. Predicting the potential need for an epicardial approach—based on the type of cardiomyopathy, previous cardiac surgery, imaging findings, and electrocardiographic markers—represents a crucial first step in building an effective treatment strategy. Within this process, mapping technology plays a pivotal role. Recent advances have profoundly transformed the way substrate evaluation is performed in epicardial ablation, and functional, high-resolution mapping techniques originally developed for endocardial procedures are now widely applicable to the epicardial surface. Methods such as ILAM, DEEP mapping, EDP mapping, and analysis of rotational activation patterns make it possible to visualize critical conduction channels and isthmus regions even when VT cannot be induced, allowing detection of arrhythmogenic substrates that might otherwise remain hidden. In addition, analyses performed during sinus rhythm—including late potential mapping, fractionation analysis, ripple mapping, early meets late lower threshold settings, multipolar mapping, and the use of peak frequency and emphasis maps—provide detailed characterization of slow conduction channels and facilitate early identification of epicardial involvement, guiding the design of optimal ablation strategies. By integrating these advanced mapping approaches with pre-procedural imaging and anatomical information, operators can more precisely define the arrhythmogenic substrate, tailor the ablation plan to each patient, and potentially improve both acute and long-term outcomes. At the same time, procedural safety must always remain a priority. Careful selection of the puncture approach, the use of adjunctive techniques such as CO_2_ insufflation or three-dimensional mapping-guided access, and preventive strategies against complications like pericarditis or coronary artery proximity are essential. Looking ahead, bipolar radiofrequency ablation, pulsed field ablation, and AI-driven computational models are expected to complement substrate-based mapping approaches and further expand therapeutic options. With these ongoing advances, epicardial ablation—supported by sophisticated mapping technologies—will continue to evolve as a cornerstone of precise, individualized arrhythmia management.
